# Reducing physician voiding cystourethrogram ordering in children with first febrile urinary tract infection: evaluation of a purposefully sequenced educational intervention

**Published:** 2018-11-12

**Authors:** Anke Banks, Susan Samuel, David Johnson, Kent Hecker, Kevin McLaughlin

**Affiliations:** 1 Department of Pediatrics, Cumming School of Medicine, University of Calgary, Alberta, Canada; 2 Department of Community Health Sciences, Cumming School of Medicine, University of Calgary, Alberta, Canada; 3 Department of Physiology and Pharmacology, Cumming School of Medicine, University of Calgary, Alberta, Canada; 4 Department of Medicine, Cumming School of Medicine, University of Calgary, Alberta, Canada

## Abstract

**Background:**

Physicians often fail to implement clinical practice guidelines. Our aim was to evaluate whether a purposefully sequenced, multifaceted educational intervention would increase physician adherence to a guideline for voiding cystourethrogram (VCUG) use following first urinary tract infection (UTI) in young children.

**Methods:**

Using a single centre, pretest-posttest design, we compared the proportion of guideline adherent VCUG orders and the VCUG ordering rate before and after three educational interventions (interactive lecture, clinical pathway, faxed reminder) selected and sequenced according to the PRECEDE (Predisposing, Reinforcing and Enabling Constructs in Educational Diagnosis and Evaluation) health promotion model.

**Results:**

One hundred and nine physicians ordered 219 VCUGs for 219 children. Following the interventions, there was an increase in the monthly proportion of adherent VCUGs ordered by pediatricians (analysis of variance (ANOVA) F(2,29) = 3.38, p = .048) and non-pediatricians (ANOVA F(2,28) = 14.71, p < .001). Also, pediatricians decreased their monthly VCUG ordering rate (linear trend incidence rate ratio 0.74, 95% confidence interval (CI) [0.54, 0.99]). Pediatricians were more likely to adhere with the guideline than were non-pediatricians (odds ratio 2.91, 95% CI [1.5, 5.5]).

**Conclusion:**

Exposure to purposefully sequenced educational interventions based on the PRECEDE model was associated with increased adherence to guideline recommendations.

## Introduction

Clinical practice guidelines synthesize best evidence to inform and direct clinical practice.^1^ Usually developed by a panel of experts and supported by a major clinical society or group, guidelines are based on a review of the empirical and clinical evidence. Despite these efforts to amalgamate best evidence, patients receive less than 55% of recommended care,^2^ and up to 20% of care may be unnecessary or even harmful.^3^

Investigators have used varying interventions to improve uptake of guideline recommendations into practice,^4-10^ with many of these strategies centered on physician continuing medical education. Utilizing the PRECEDE (Predisposing, Reinforcing and Enabling Constructs in Educational Diagnosis and Evaluation) health promotion model developed by Green and Kreuter,^11^ Davis *et al*. have suggested that physician educational interventions may be categorized into predisposing, reinforcing, and enabling types.^12-14^ Predisposing educational interventions (e.g., printed educational materials, large-grouplectures) target knowledge, beliefs and attitudes and are designed to motivate behaviour change.^13,14^ Enabling interventions facilitate the process of behaviour change using such resources as locally developed clinical pathways or hands-on workshops.^13,14^ Finally, reinforcing interventions such as reminders assist decision-making in the immediate work environment and help sustain behaviour change over time.^13,14^

Davis *et al*. further suggested that choosing an intervention category to match physician readiness for behaviour change may optimize knowledge translation.^13,14^ This may be exemplified using Prochaska’s Stages of Change model.^15^ Physicians in the Prochaska’s Pre-contemplation stage may be ignorant of a guideline update and may benefit from a predisposing educational intervention that increases awareness. On the other hand, those in the Contemplation, Preparation, or Action phases may be ready to incorporate guideline recommendations and may find an enabling intervention helps them follow through with a planned behaviour change. Finally, reinforcing interventions may best serve physicians in the Maintenance phase who desire ongoing adherence to recommendations and avoidance of relapse.

Single educational interventions have not uniformly led to substantial behaviour change.^4-10^ However, a knowledge translation strategy that purposefully sequences predisposing, enabling, and reinforcing educational interventions may target physicians in various Stages of Change and thus have more impact. The primary objective of this study was to evaluate whether this form of multifaceted educational intervention would increase uptake of the recently revised American Academy of Pediatrics (AAP) guideline regarding voiding cystourethrogram (VCUG) use in first febrile urinary tract infection (UTI) in 2-24 month old children.^16^ In contrast to the previous guideline,^17^ this update recommends selective, rather than routine, use of VCUGs following the first UTI. We hypothesized that if our intervention improved guideline uptake, then after the intervention, the proportion of guideline adherent VCUGs would increase and the total number of VCUGs ordered would decrease. Further, we predicted that the group receiving three interventions (pediatricians) would be more likely to be adherent than those receiving one intervention (non-pediatricians). Our secondary objective was to determine if specific physician and patient characteristics influenced adherent ordering.

## Methods

### Study design and setting

We conducted a prospective interventional study with a pretest-posttest design at the Alberta Children’s Hospital, an urban tertiary care hospital in Calgary, Canada. This hospital provides pediatric services to a catchment area of 2.5 million people and performs the majority of pediatric VCUGs in Southern Alberta.

### Compliance with ethical standards

We obtained ethics approval from the Conjoint Health and Research Ethics Board, University of Calgary, Calgary, Canada. Informed consent was waived as knowledge of the study might influence physician behaviour. Instead, we sent a debrief letter to all physicians who received the reinforcing intervention. No physician requested data removal.

### VCUG ordering adherence

We defined VCUG ordering adherence using AAP guideline recommendations. An adherent VCUG request was a VCUG ordered for a 2-24 month old child with a first UTI preceded by 1) a positive urine culture (defined as >10^7^ colony forming units per liter for a typical single uropathogen) and 2) an abnormal renal and bladder ultrasound (RBUS). We defined a non-adherent VCUG request as a VCUG ordered without both of these elements.

### Intervention

Using the PRECEDE health promotion model and Prochaska’s Stages of Change,^11,13-15^ we developed a multifaceted implementation strategy with predisposing, enabling, and reinforcing components to increase physician VCUG ordering adherence.

The predisposing intervention was a grand rounds presentation of the UTI guideline that was video-conferenced to regional hospitals and pediatric clinics in Southern Alberta. On-site audience members could participate in an interactive lecture component using an audience response system.

The enabling intervention was a clinical pathway adapted from the AAP guideline by local pediatric infectious diseases, nephrology, emergency medicine, and hospitalist physicians (Supplementary Material 1). It was reviewed by physicians from the divisions of pediatric urology and radiology, then introduced at the grand rounds seminar. To ensure point-of-care access, a PDF version was posted on two internal hospital websites and a publicly accessible external website and was emailed to pediatric nephrologists, hospitalists, and chief residents.

The reinforcing intervention was a standardized form faxed to all physicians who ordered a non-adherent VCUG from the hospital diagnostic imaging department (Supplementary Material 2). It listed the AAP guideline’s imaging recommendations and required that physicians acknowledge these recommendations and return the form in order to have the non-adherent VCUG performed. It also provided a space for physicians to enter a reason if they chose to continue with the VCUG request.

Adherence was reassessed after the form was faxed. If the form was returned indicating an adherent reason for ordering, the VCUG request was re-classified as adherent. If the form was not returned, the VCUG was cancelled and the request was considered post-reminder adherent. If the form was returned requesting the VCUG without a reason or with a non-adherent indication, the request was considered post-reminder non-adherent.

### Study population

Pediatricians and non-pediatricians were examined separately. The 263 general and sub-specialist pediatricians in Calgary had the potential to receive all three interventions. However, non-pediatricians were only targeted to receive the reinforcing component.

### Study phases

The AAP guideline was published in September 2011. We allotted two months for online and print distribution of the guideline. We defined the next 24 months (December 1, 2011-November 30, 2013) as the Pre-intervention Phase. We defined the Predisposing/Enabling Phase as the five months (December 1, 2013-April 30, 2014) following the grand rounds presentation and introduction of the clinical pathway. We defined the Reinforcing Phase as the three months (May 1-July 31, 2014) after the introduction of the reminder intervention.

### Inclusion criteria

In all three phases, we included VCUGs ordered to investigate possible vesicoureteral reflux in children 2-24 months old at the time of the last UTI, and when either of these criteria were met: 1) a preceding positive urine culture, or 2) in the absence of a documented positive urine culture, either “urinary tract infection” or “pyelonephritis” was documented as the VCUG clinical indication on the requisition or patient chart.

### Data sources

We used the IMPAX (Agfa HealthCare) Picture Archive and Communications System to list all VCUGs performed at the hospital’s diagnostic imaging department during the Pre-intervention and Predisposing/Enabling Phases. During the Reinforcing Phase, we determined eligible VCUGs by screening all VCUG requests for 2-24 month olds received by the diagnostic imaging department. We obtained clinical information from Calgary Lab Services, IMPAX, review of paper charts, and the hospital’s electronic health record (Allscripts Sunrise Clinical Manager™). We used the publicly accessible College of Physicians and Surgeons of Alberta website (www.cpsa.ab.ca) to collect physician demographic information.

### Outcome variables

Our primary outcome was the monthly proportion of adherent VCUGs ordered by physicians for 2-24 month old infants with a first UTI during each intervention period. Other outcomes included the monthly ordering rate of VCUGs and the proportion of adherent VCUGs at Reinforcing Phase screening compared to immediately after the reminder. We also determined physician and patient characteristics associated with adherence.

### Statistical analysis

We performed statistical analysis with Stata SE 12 (StataCorp™) and set the significance level at alpha = 0.05. We used descriptive statistics (proportions or median and interquartile range (IQR)) to report ordering physician characteristics. We compared the characteristics of pediatricians and non-pediatricians ordering VCUGs using the chi-square test of proportion, Wilcoxon rank-sum test or unpaired 2-sample *t*-test as appropriate. For our primary outcome, we reported the proportion of adherent VCUGs ordered by pediatricians during each time period. These proportions were based on a monthly average for each time period. We compared means across the time periods using analysis of variance (ANOVA) followed by Fisher-Hayter pairwise comparison of means. We performed a similar analysis on non-pediatrician ordering using ANOVA and Bonferroni pairwise comparison of means. We also describe the monthly VCUG ordering rate for pediatricians and non-pediatricians during each intervention phase. We used Poisson regression to test for a change over time and reported outcomes as incidence rate ratios (IRRs). We used McNemar’s test to compare the immediate change in adherence in each physician group after exposure to the reminder. We used multivariate logistic regression to assess physician (physician specialty, sex, years in practice, practice location) and patient (premature birth, age, non-*Escherichia coli* uropathogen, hospitalization with UTI) characteristics associated with adherence. We also included dummy variables for predisposing/enabling intervention exposure and reinforcing intervention exposure. We used backward elimination to remove non-significant predictors (*p* ≥ .05) with rechecking of the model at each step.

## Results

### Population characteristics

We analyzed 219 VCUGs ordered on 219 children (Pre-intervention, n=177; Predisposing/Enabling, n = 27; and Reinforcing, n = 15). Of the 109 physicians ordering VCUGs, just over half (51.4%) were female with a median of 1 VCUG (IQR 1, range 1-17) ordered per physician (Table 1). There were 68 (62.4%) pediatricians and 41 (37.6%) non-pediatricians among ordering physicians (Table 2). As a group, pediatricians ordered more VCUGs per month (4.5, standard deviation (SD) 2.5) than non-pediatricians (2.3, SD 1.5, p < .001).

**Table 1:** Characteristics of physicians ordering VCUGs by study phase

Characteristic	Physicians, n (%)^a^			
	Overall (n = 109)	Pre-intervention (n = 94)	Predisposing/Enabling (n = 22)	Reinforcing (n = 13)
Female sex	56 (51.4)	47 (50.0)	8 (36.4)	9 (69.2)
**Physician specialty**				
Pediatrician	68 (62.4)	60 (63.8)	15 (68.2)	8 (61.5)
Non-pediatrician	41 (37.6)	34 (36.2)	7 (31.8)	5 (38.4)
Years since graduating medical school, median (IQR, range)	16.2 (15.4, 3.4-46.4)	16.7 (16.1, 3.4-44.7)	15.7 (14.1, 3.6-46.8)	14.0 (10.9, 4.9-29.9)
Unable to assess	1 (0.9)	1 (1.1)	0 (0)	0 (0)
Urban location of practice	102 (93.6)	89 (94.7)	20 (90.9)	13 (100)
Number of VCUGs ordered per physician, median (IQR, range)	1 (1, 1-17)	1 (1, 1-12)	1 (0, 1-3)	1 (0, 1-3)

a Unless otherwise noted

**Table 2:** Comparison of physicians ordering VCUGs over total study period

Characteristic	Pediatricians (n = 68)	Non-pediatricians (n = 41)	*p* value
Female sex, percentage	54.4	46.3	.41^a^
Years since graduating medical school, median (IQR, range)	16.3 (16.5, 4.9-40.7)	16.2 (14.8, 3.4-46.4)	.80^b^
Urban location of practice, percentage	100	82.9	<.001^a^
Total number of VCUGs ordered	144	75	<.001^a^
Number of VCUGs ordered per physician, median (IQR, range)	1 (2, 1-9)	1 (0, 1-17)	.002^b^
Monthly ordering of VCUGs, mean (SD, range)	4.5 (2.5, 1-12)	2.3 (1.5, 0-6)	<.001^c^

a chi-square test of proportion

b Wilcoxon rank-sum test

c t-test

### Guideline adherence

**Figure 1:**
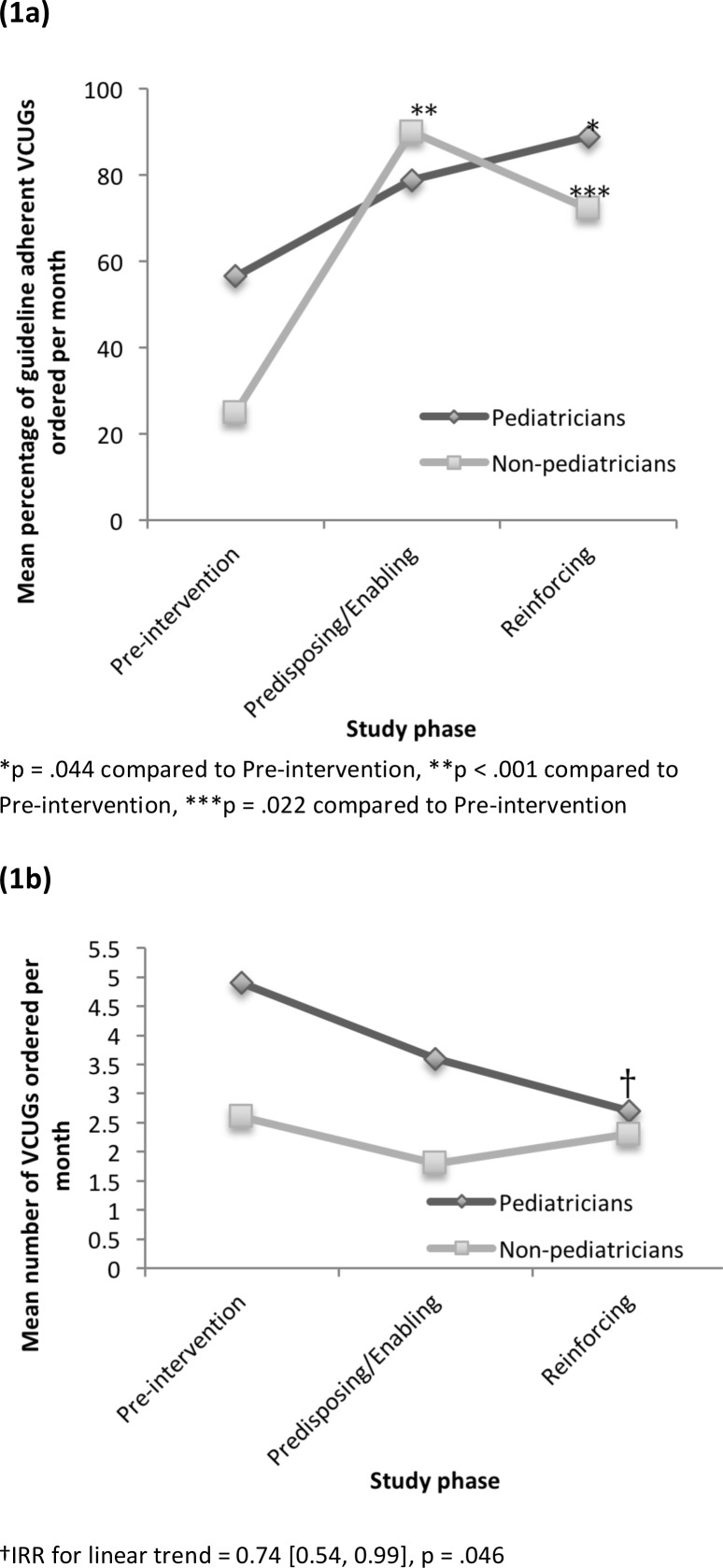
Change in VCUG ordering adherence (1a) and rate (1b) over the study period

The average monthly proportion of adherent VCUGs ordered by pediatricians significantly increased from 56.6% (SD 25.0) in the Pre-intervention Phase to 78.8% (SD 27.5) and 88.9% (SD 19.2) after the Predisposing/Enabling and Reinforcing Phases as determined by one-way ANOVA (F(2,29) = 3.38, p = .048) (Figure 1a). Pediatricians ordered a significantly higher monthly proportion of adherent VCUGs after the Reinforcing Phase compared to the Pre-intervention Phase (p = .044).

Non-pediatricians also demonstrated a significant increase in the average monthly proportion of adherent VCUGs ordered over the study period as determined by one-way ANOVA (F(2,28) = 14.71, p <.001). Non-pediatrician monthly adherence was significantly higher after both the Predisposing/Enabling Phase (90.0% (SD 22.4), p < .001) and Reinforcing Phase (72.2% (SD 25.4), p = .022) compared to the Pre-intervention Phase (25.0% (SD 27.4)).

The pediatrician monthly ordering rate of VCUGs decreased from 4.9 (SD 2.6) to 3.6 (SD 2.2) to 2.7 (SD 0.6) for Pre-intervention, Predisposing/Enabling, and Reinforcing Phases, respectively (Figure 1b). This represents an incidence rate ratio (IRR) of 0.73, 95% CI [0.45, 1.20] during the Predisposing/Enabling Phase and 0.54, 95% CI [0.27, 1.11] during the Reinforcing Phase, with an overall significant linear trend (IRR for linear trend = 0.74, 95% CI [0.54, 0.99], p = .046). For non-pediatricians, the monthly ordering rates were 2.6 (SD 1.6), 1.8 (SD 1.3), and 2.3 (SD 0.58) for the Pre-intervention, Predisposing/Enabling and Reinforcing Phases, respectively. The corresponding IRRs were 0.73, 95% CI [0.36, 1.48] and 0.95, 95% CI [0.43, 2.08] in the Predisposing/Enabling and Reinforcing Phases, respectively, with no evidence of a linear trend (IRR for linear trend = 0.91, 95% CI [0.63, 1.32], p = .63).

During Reinforcing Phase screening of VCUG requests, 87.5% (7/8) of pediatrician requests were adherent with no change following the faxed reminder. Conversely, 28.6% (2/7) of non-pediatrician VCUG requests were adherent at screening. Following the reminder, although not statistically significant, adherence increased to 71.4% (5/7) (p = .083).

### Factors influencing VCUG ordering

We determined that the odds of an adherent VCUG were 2.91 times greater among pediatricians than non-pediatricians (Table 3). Those exposed to the predisposing/enabling intervention or reinforcing intervention had increased odds of adherence, whereas female physicians had decreased odds of adherence.

**Table 3:** Predictors of VCUG ordering adherence

Predictor	Odds ratio	95% CI	*p* value
**Pediatrician**	2.91	1.54, 5.51	.001
**Female physician**	0.44	0.24, 0.80	.007
**Predisposing/enabling intervention exposure**	5.28	1.88, 14.78	.002
**Reinforcing intervention exposure**	6.49	1.70, 24.81	.006

The following predictors were considered but removed from the final model: physician characteristics (years in practice and practice location) and patient characteristics (premature birth, age, non-*Escherichia coli* uropathogen, hospitalization with UTI).

## Discussion

We implemented a multifaceted educational intervention that specifically targeted pediatricians. We found that after the intervention, pediatricians ordered a greater proportion of VCUGs adherent to the AAP guideline compared to before the intervention. As well, their monthly VCUG ordering rate substantially declined over the study period. Non-pediatricians, on the other hand, were only intentionally exposed to the reinforcing intervention. Their ordering adherence was markedly improved after this intervention compared to pre-intervention, but their monthly ordering rate did not significantly decline.

Several systematic reviews have investigated the usefulness of multifaceted interventions.^5,6,8,18^ Although multifaceted interventions do not consistently perform better than single interventions,^6,8,18^ they are more likely to be effective if they have a sound theoretical foundation based on an analysis of barriers to change.^5,6^ As well, a systematic review highlighted that the combination of predisposing, enabling, and reinforcing educational interventions achieved better results than using one or two of these components alone.^19^ Thus we purposefully chose and sequenced three educational interventions targeting different Stages of Change. Similar to other groups,^19,20^ we found success with this educational intervention strategy.

As part of our study design, we did not intentionally provide non-pediatricians with the grand rounds or clinical pathway interventions. Yet, they demonstrated an increase in monthly ordering adherence during the Predisposing/Enabling Phase compared to the Pre-intervention Phase. This may reflect guideline uptake from other sources. It may also reflect an unintended extension of the clinical pathway enabling intervention. Although pediatric urologists (classed in the non-pediatrician group) were not targeted to receive the clinical pathway intervention, the group reviewed and endorsed the pathway prior to its distribution. Becoming involved in the guideline adaptation process can create a sense of guideline ownership^21^ and may have improved their willingness to use the recommendations in the months that followed their review.

Reminders generally lead to a moderate improvement in physician performance.^6,10,22^ Furthermore, physician performance is improved when a reminder is added to educational meetings and materials.^6^ In our study, pediatrician and non-pediatrician monthly ordering adherence was significantly higher in the Reinforcing Phase compared to baseline, but neither group had a significant change in adherence between the Predisposing/Enabling Phase and Reinforcing Phase. We examined this further by assessing the change in adherence directly associated with the reminder. Of the screened VCUG requests, the majority of non-pediatrician requests were non-adherent. After sending a reminder, ordering adherence increased almost 2.5 fold. In contrast, 87.5% of pediatrician VCUG requests were adherent and therefore did not trigger a reminder intervention. The high pediatrician adherence in this phase may instead reflect the endurance of the predisposing intervention or ongoing use of the clinical pathway enabling intervention. On the other hand, the reminder seemed to have a greater impact on non-pediatricians, where it was the only planned intervention.

In contrast to previous studies of guideline adherence in test ordering,^23-27^ we specifically considered physician characteristics that may have influenced VCUG ordering. We determined that pediatricians were 2.91 times more likely to order adherent VCUGs than non-pediatricians. This may be because they received the sequenced three-part educational intervention rather than a single intervention. On the other hand, it may simply be that pediatricians are more familiar with pediatric guidelines than non-pediatricians. The volume of new research makes guideline awareness and familiarity an ongoing barrier for any physician,^28^ let alone a general practitioner. Furthermore, physicians express confidence in guidelines developed by their own specialty society,^29^ potentially leading to improved adherence.

We also found that female physicians were less likely to order adherent VCUGs. This was a surprising finding given others demonstrating that female physicians have superior guideline knowledge,^30^ adherence,^31^ and participation in maintenance of certification^32^ than male physicians. However, previous investigations have often centered on interventions to increase test ordering when it was appropriate (e.g., pap smears^33^), rather than reducing test ordering. In fact, female physicians are more likely to order cancer screening^34^ and general investigations compared to their male counterparts.^35^ Thus, one explanation for the lower adherence by female physicians is that it may be more difficult to reduce test ordering in physicians who are prone to ordering tests.

This study has several limitations. The new guideline recommends selective VCUG ordering with a first UTI. Although we demonstrated an overall decrease in the number of VCUGs ordered, we did not record the total number of children with first UTIs during our study period. Thus, we may have underestimated the decline in VCUG ordering. Another limitation is that only 15 VCUGs were ordered during the reminder intervention period. This led to wide confidence intervals in the logistic regression analysis and reduced the precision of our results. The pretest-posttest design is limited in that it does not account for factors outside the planned intervention that could alter physician VCUG ordering behaviour. We also did not account for clustering of VCUG ordering amongst physicians. As well, our study design did not allow us to assess which pediatricians were exposed to the predisposing and enabling interventions. Thus, it is possible that adherence may have improved without exposure to any or all parts of the intervention.

In addition to the ultrasound criteria, the AAP guideline recommends that with a first febrile UTI, VCUGs could be ordered for children with “complex clinical circumstances.” Because this is a subjective assessment by the ordering physician, we did not devise criteria for its inclusion in our definition of guideline adherence. This may have led to an underestimation of guideline adherence. However, because patient characteristics were similar over the three study phases (data not shown), this underestimation may have been equally present and may not have affected overall results. Finally, although the interventions were chosen to overcome barriers to guideline uptake known to be present in many tertiary care settings, the study was only conducted at a single site. This may limit generalizability of results.

### Conclusion

Guideline adherence improved following implementation of our unique knowledge translation strategy with sequenced predisposing, enabling, and reinforcing educational interventions. Importantly, adherence was associated with physician specialty and sex. These findings this should inform the development of physician educational interventions within continuing medical education programs. Additionally, using interventions that target multiple Stages of Change should be considered a promising addition to present strategies promoting knowledge translation and physician behaviour change.
